# Hepatic Stellate Cells and microRNAs in Pathogenesis of Liver Fibrosis

**DOI:** 10.3390/jcm5030038

**Published:** 2016-03-16

**Authors:** Mio Kitano, P. Mark Bloomston

**Affiliations:** 1 Division of Surgical Oncology, Department of Surgery, Wexner Medical Center, The Ohio State University Wexner Medical Center, Columbus, OH 43210, USA; kitmio@gmail.com; 2 21st Century Oncology, 4571 Colonial Blvd., Suite 210, Fort Myers, FL 33966, USA

**Keywords:** liver disease, microRNA, hepatic stellate cell, liver fibrosis

## Abstract

microRNAs (miRNAs) are small non-coding RNAs that regulate gene expression by either blocking translation or inducing degradation of target mRNA. miRNAs play essential roles in diverse biological and pathological processes, including development of hepatic fibrosis. Hepatic stellate cells (HSCs) play a central role in development of hepatic fibrosis and there are intricate regulatory effects of miRNAs on their activation, proliferation, collagen production, migration, and apoptosis. There are multiple differentially expressed miRNAs in activated HSCs, and in this review we aim to summarize current data on miRNAs that participate in the development of hepatic fibrosis. Based on this review, miRNAs may serve as biomarkers for diagnosis of liver disease, as well as markers of disease progression. Most importantly, dysregulated miRNAs may potentially be targeted by novel therapies to treat and reverse progression of hepatic fibrosis.

## 1. Introduction

Hepatic fibrosis ensues secondary to chronic hepatic injury and inflammation and some of the common etiologies are viral and autoimmune hepatitis, alcohol consumption, nonalcoholic steatohepatitis (NASH), metabolic diseases leading to copper or iron overload, toxins, and biliary obstruction [1,2]. It is characterized and accompanied by deposition of extracellular matrix (ECM) with distortion of normal hepatic parenchyma, eventually leading to dense fibrosis, cirrhosis, and development of portal hypertension [3]. Hepatic stellate cells (HSCs) are the main ECM-producing cells and there has been a dramatic increase in the number of publications related to HSCs in the past two decades, indicating increased recognition of their versatile functions, as well as increased interest in utilizing them for therapeutic applications to treat and reverse hepatic fibrosis [4].

microRNAs (miRNAs) are small non-coding RNAs first described by Ambros *et al.*, in 1993 [5]. They discovered a small non-coding transcript of lin-4 gene that interacted with lin-4 messenger RNA (mRNA) and regulated translation and controlled post-embryonic development of *c. elegans* [5]. They are approximately 20–27 nucleotides long and function as regulatory molecules by binding to target mRNA in the 3′-untranslated region (3′-UTR) leading to either repression of mRNA translation or promoting degradation [6,7]. Since their discovery, there have been approximately 1880 miRNAs identified in *homo sapiens*, with each miRNA thought to target on average 200 transcripts; thus regulating up to one third of human genes [8,9,10]. miRNAs play an essential role in gene expression and regulate various biological processes such as cell differentiation, apoptosis, metabolism, and endocrine function [11,12,13,14]. Furthermore, aberrant expression is associated with various pathological processes, including carcinogenesis, neurodegenerative, and heart diseases [15,16,17]. The ability of a single miRNA to regulate multiple gene expressions makes them an appealing target for therapeutic intervention. Additionally, their potential role as prognostic indicators and diagnostic markers has been increasingly studied and recognized [18,19,20]. This review aims to summarize current knowledge of microRNAs and HSCs in the development of hepatic fibrosis and their potential role as therapeutic targets and biomarkers.

## 2. Biogenesis of microRNA

miRNAs are transcribed in the nucleus by RNA polymerase II into primary miRNA (pri-miRNA) consisting of one or more hairpin structures before they are cleaved into a 60–110 nucleotide base precursor miRNAs (pre-miRNA) by RNAse III enzyme named Drosha and dsRNA-binding domain protein (dsRBD) named DGCR8/Pasha [21,22]. pre-miRNAs are transported into the cytoplasm by exportin-5 through a Ran-guanine-triphosphate (GTP)-dependent pathway and they undergo further cleavage by another RNase III named Dicer and dsRBD protein TRBP/PACT [23]. Lastly, single-stranded mature miRNAs interact with Argonaute (ARO) protein and is then degraded in the RNA-induced silencing complex (RISC), which in turn modulates mRNA degradation and repression or activation of translation [24].

## 3. The Role of Hepatic Stellate Cells in the Development of Liver Fibrosis

**Figure 1:**
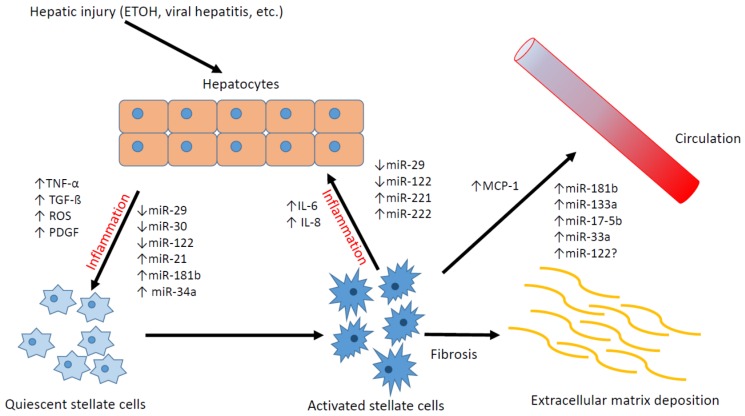
Relationship between hepatic stellate cells, hepatocytes, and microRNAs.

HSCs are liver-specific mesenchymal cells located in the perisinusoidal space known as the space of Disse [1]. Their quiescent forms comprise about 5%–8% of all cells in the liver [25,26]. They play a central role in hepatic development, regeneration, lipoprotein and retinoid metabolism, immune regulation in normal livers, and fibrogenesis in response to hepatic injury [4,26,27]. In response to injury, they become activated and lose their normal role of retinoid storage. They proliferate and secrete ECM proteins such as collagen, glycoprotein, and proteoglycans leading to hepatic fibrosis [2,28,29]. Fibrosis leads to impaired hepatocyte function and increased intrahepatic resistance to blood flow leading to portal hypertension [2]. HSC activation is promoted by tumor necrosis factor-α (TNF-α), transforming growth factor-β and -α (TGF-β and -α), and reactive oxygen species (ROS) produced by apoptotic hepatocytes and Kuppfer cells during hepatic injury [30]. HSCs then transform into myofibroblast-like cells from their quiescent form [2,28]. Activated HSCs, in turn, secrete pro-inflammatory cytokines (e.g., Interleukin-6, Interleukin-8, monocyte chemoattractant protein-1) further perpetuating an inflammatory state (Figure 1) [28]. There are conflicting data regarding the origin of HSCs. Some claim HSCs originate from hepatic epithelial cells during hepatic injury through epithelial-mesenchymal transition (EMT) [31], while other studies suggest HSCs originate from bone-marrow derived mesenchymal cells [32,33]. In animal models, the main distinctive feature in activated HSCs is the presence of α-smooth muscle actin (α-SMA), a myofibroblast marker, while quiescent HSCs express desmin and glial fibrillary acidic protein [26,34].

There are various causes of chronic hepatic injury and the mainstay method to halt progression of fibrogenesis is to remove the inciting liver-injuring etiology [2,28]. In addition to halting the progression of fibrosis by removing the inciting etiology, HSC apoptosis has been demonstrated to reverse hepatic fibrosis [2,35,36,37]. Since chronic liver diseases and fibrosis are linked to the development of hepatocellular carcinoma (HCC), there has been increasing interest in studying HSCs as potential targets for treatment and prevention of hepatic fibrosis and development of HCC [2,35,36,37,38].

## 4. Differentially Expressed MicroRNAs in Activated Hepatic Stellate Cells

There are several studies that have analyzed miRNA expression profiling in quiescent and activated HSCs in order to elucidate their regulatory pathways (Table 1) [39,40,41,42,43,44,45,46]. The majority of miRNA expression profilings were done on animal models and there were also heterogeneity in their method of HSC activation. *In vitro* culture activation was utilized in some studies [41,42,43,44] and some utilized *in vivo* activation method by harvesting the RNA from animals treated with hepatotoxic agents [39,45,46].

*In vitro* or culture activation of HSC is based on the knowledge that freshly harvested HSCs are considered “quiescent” with lack of expression of α-SMA [34,48]. After several days (5–14 days) of culture, they change their morphology and produce α-SMA [34,43,48]. miRNA expression profiling in *in vitro* activated HSCs showed down-regulation of members of the miR-17-92 cluster (miR-19a, -19b, -92a) [43]. Comparative bioinformatics analysis of microarrays comparing quiescent and *in vitro* activated HSCs demonstrated 21 miRNAs, 12 up-regulated (miR-874, miR-29C*, miR-501, miR-349, miR-325-5p, miR-328, miR-138, miR-143, miR-207, miR-872, miR-140, and miR-193) and 9 down-regulated (miR-341, miR-20b-3p, miR-15b, miR-16, miR-375, miR-122, miR-146a, miR-92b, and miR-126) miRNAs [41,42]. This group further analyzed the data and found 25 pathways that are significantly regulated by these miRNAs [41,42]. Maubach *et al.*, demonstrated 16 up-regulated and 26 down-regulated miRNAs in *in vitro* activated HSCs.

There are several miRNA expression profiling performed in *in vivo* activation model. As the name implies, expression profiling is done on HSCs isolated from animals exposed to hepatotoxic agents. For example, there were 11 significantly up-regulated miRNAs in a carbon tetrachloride (CCL_4_)-induced mouse liver fibrosis model [45]. Du *et al.*, performed miRNA microarray analysis in non-alcoholic steatohepatitis model in C57BL/6J mice using methionine-choline deficient diet and identified 19 up-regulated and 18 down-regulated miRNAs in liver with fibrosis [39]. Among those dysregulated miRNAs, miR-146a-5p was found to be the most significantly down-regulated miRNA [39]. Another microarray performed in *in vivo* CCL_4_-induced hepatic fibrosis mouse model demonstrated significant down-regulation of miR-29 family, and they validated their result on human samples [46].

Cheung *et al.*, performed microarray analysis of liver biopsy samples obtained from patients with nonalcoholic steatohepatitis (NASH) and demonstrated 46 dysregulated miRNAs (23 up-regulated and 23 down-regulated) [47]. A high-throughput sequencing on human liver biopsy samples obtained from 15 individuals with normal liver and 15 individuals with severe nonalcoholic fatty liver disease (NAFLD) identified total of 75 significantly differentially expressed miRNAs, including 30 up-regulated and 45 down-regulated miRNAs [40].

The difference and the heterogeneity of dysregulated miRNA profile between studies are likely secondary due to the different methods of HSC activation, etiology of hepatic injury, and variation between different species.

## 5. The Role of MicroRNAs in Liver Fibrosis

There are various miRNAs implicated in the development of liver fibrosis, with many of them affecting different steps of fibrogenesis (HSC activation, HSC proliferation, ECM maturation, *etc.*). miRNAs involved in hepatic fibrosis can be broadly categorized into pro-fibrotic or anti-fibrotic miRNAs, where pro-fibrotic miRNAs are up-regulated and anti-fibrotic miRNAs are down-regulated during fibrogenesis (Table 2 and Table 3).

### 5.1. microRNAs Regulate HSC Activation

miR-29 is one of the most studied and cited miRNAs in the development of hepatic fibrosis. Both hepatocytes and HSCs produce similar amounts of miR-29 in the liver, but its levels are significantly down-regulated in patients with chronic HCV infection [47]. It is an anti-fibrogenic miRNA and it plays an essential role in HSC activation. Transforming growth factor (TGF)-β, a key fibrogenic cytokine, is a powerful activator of HSCs. TGF-β down-regulates miR-29 and promotes a profibrogenic state by activating HSC and enhancing deposition of ECM [67,68]. Conversely, hepatocyte growth factor (HGF), which reduces TGF-β mRNA levels, up-regulates miR-29 and promotes an anti-fibrogenic state [68]. Kwiencinski *et al.*, demonstrated loss of miR-29 in activated HSC during myofibroblastic transition and with that there was increased expression of profibrinogenic genes such as platelet-derived growth factor (PDGF)-B and insulin-like growth factor (IGF)-I [69]. Wang *et al.*, demonstrated miR-29 attenuated HSC activation and induced their apoptosis via inhibition of PI3K/AKT pathway in CCL4-induced fibrosis mouse model with PI3K and AKT3 knockdown and *in vitro* transfection on LX-1 and HSC-T6 cell lines [94]. miR-29 and -29a also regulate HSC activation via histone deacetylases (HDACs). Mannaerts *et al.*, demonstrated HDAC4 activated HSC via lysyl oxidase expression and they showed HSC activation was suppressed with pharmacological inhibitor of HDAC and with that there was strong up-regulation of miR-29 [95]. Huang *et al.*, demonstrated similar findings with up-regulation of miR-29a in activated HSCs through inhibition of HDAC4, which in turn abrogated cholestatic liver fibrosis in mice after bile duct ligation [71]. Sekiya and colleagues demonstrated transfection of miR-29 precursor blunted the expression of genes responsible for HSC activation via suppression of proto-oncogene c-fos mRNA [96].

miR-21 is one of the predominantly up-regulated miRNAs during fibrogenesis. Stimulation of LX-2 cells with platelet-derived growth factor (PDGF)-BB, a known pro-fibrogenic protein, significantly stimulated expression of α1 collagen mRNA synthesis and protein expression of α-SMA while simultaneously increasing miR-21 levels [51]. Down-regulation of miR-21 by transfecting LX-2 cells with anti-miR-21 prevented PDGF-BB-induced LX-2 activation while its up-regulation enhanced LX-2 activation [51]. Furthermore, there was inverse correlation with miR-21 levels with phosphatase and tension homolog deleted on chromosome 10 (PTEN) expression. In other words, overexpression of miR-21 led to decreased expression of PTEN and subsequent activation of Akt. The authors concluded that miR-21 might play a role in HSC activation via the PTEN/Akt signaling pathway [51]. miR-21 maintains its up-regulation via a miR-21/programmed cell death protein 4/activation protein-1 (miR-21/PDCD4/AP-1) feedback loop [52]. Disrupting this feedback loop by using either miR-21 or AP-1 inhibitors significantly diminished fibrogenesis while blocking PDCD4 enhanced fibrogenesis in HSCs [52]. Additionally, miR-21 modulated extracellular signal-regulated kinase 1 (ERK1) signaling and EMT during fibrogenesis by regulating hepatocyte nuclear factor 4α (HNF4α) and sprouty2 (SPRY2) expression [53]. Inhibition of miR-21 suppressed ERK1 signaling and inhibited HSC activation while blocking EMT in TGFβ1-treated hepatocytes. Conversely, stimulation of miR-21 enhanced ERK1 signaling in HSCs and induced hepatocyte EMT by targeting HNF 4α and SPRY2 [53].

Based on literature, miRNAs control various signaling pathways in order to regulate HSC biology and activation. Some examples are PTEN/Akt, SMAD family, PPARγ, EMT/ERK1, and NF-κB signaling pathways. miR-181b, in addition to miR-21, is involved in PTEN/Akt signaling pathway [97]. Similar to miR-21, miR-181b is up-regulated in liver fibrosis by TGF-β1 and it promotes HSC proliferation and collagen deposition and its pro-fibrotic effect was blocked by Akt inhibitor LY294002 [97]. Examples of miRNAs involved in PPARγ signaling pathway are miR-33a, 34a and -34c. There is an inverse correlation between miR-33a, 34a and -34c with expression of PPARγ during HSC activation [55,57]. It was demonstrated that miR-33a, miR-34a and -34c inhibitors up-regulated expression of PPARγ and in turn expression of α-SMA was down-regulated [55,57]. Examples of miRNAs involved in NF-κB signaling pathway are miR-221, -222, and-126. Feng *et al.*, studied the regulatory pathway of NF-κB, a critical mediator of inflammatory response and promoter of HSC activation in the liver, in relation to miR-126 [59]. They demonstrated miR-126 over-expression significantly suppressed NF-κB inhibitor alpha (IκBα) at the post-transcriptional level, leading to increased NF-κB expression [59]. Additionally, miR-126 over-expression increased TGF-β1 and collagen I mRNA expression, perpetuating further fibrotic state [59]. miR-221 and -222 are up-regulated in activated LX-2 HSC cells and their expression was reduced by NF-κB inhibitor and conversely their expression was enhanced by pro-fibrotic proteins TGFα and tumor necrosis factor (TNF)-α [64]. SMAD families are downstream transcription factors for TGF-β1 and miR-146a and -31 modulate SMAD 4 and SMAD 3, respectively [54,82]. miR-30 is down-regulated in CCL_4_-induced fibrotic liver and its overexpression inhibited TGF-β1 via a negative feedback loop by targeting Krüppel-like factor 11 (KLF11), an early response transcription factor, which potentiates TGF-β/Smad signaling by suppressing the transcription of inhibitory gene Smad7 [75]. Another miRNA involved in TGF-β/Smad signaling is miR-17-5p [50]. miR-17-5p level was increased in both CCL4-induced fibrotic liver in mice and in HSCs treated with TGF-β1 and was associated with TGF-β1-induced expression of type I collagen and α-SMA. Conversely, inhibition of miR-17-5p significantly diminished expression of type I collagen and α-SMA [50].

Although most of the studies on HSCs are performed *in vitro* and in animal models, there are some studies that have evaluated miRNAs in patient samples. For example, Expression of miR-144 and TGF-β1 levels were quantified in human fibrotic and normal liver tissues and there was significant down-regulation of miR-144, which inversely correlated with TGF-β1 levels. The authors concluded that miR-144 could be a novel regulator of TGF-β1-induced activation of HSC during hepatic fibrogenesis [81]. miR-155 is significantly down-regulated in activated HSCs in patients with cirrhosis and it regulates HSC activation via EMT and ERK1 signaling pathway [85]. miR-483 is down-regulated in HCC samples as well as in rat liver fibrosis models. Enhancing its expression suppressed the activation of HSCs *in vitro* through suppression of TGF-β1 stimulation of HSCs by negatively targeting two pro-fibrotic factors, platelet-derived growth factor-β (PDGF-β) and tissue inhibitor of metalloproteinase 2 (TIMP2) [93].

Oxidative stress is thought to play a role in HSC activation. Yang *et al.*, showed miR-200a targeted Kelch-like ECH-associated protein 1 (Keap1), a regulator of antioxidant stress response [87]. Keap1 regulates Nuclear factor-erythroid-2-related factor 2 (Nrf2), which is an important transcription factor responsible for inducing phase II detoxifying and antioxidant enzymes [87]. They demonstrated enhancement of miR-200a expression using mimics restored Nrf2-dependent NQO1 gene expression and inhibited TGF-β1-idependent growth of HSCs. They concluded miR-200a regulates Keap1 expression, restores Nrf-dependent antioxidant pathway, and reduces progression of hepatic fibrosis [87].

Yan *et al.*, showed miR-34a promoted hepatic stellate cell activation via targeting acyl-CoA synthetase long-chain family member 1 (ACSL1) with an inverse relationship between miR-34a and ACSL1 [58]. Inhibition of miR-34a, which is up-regulated in dimethylnitrosamine (DNS)-induced hepatic fibrosis rat model, increased expression of ACSL1 and lowered α-SMA [58]. miR-214-5p plays a role in HSC activation via Twist-1 pathway and its overexpression in LX-2 HSC cell lines led to increased levels of fibrosis-related genes, such as matrix metalloproteinase (MMP)-2, MMP-9, α-SMA, and TGF-β1 [63]. Over-expression of miR-146a-5p, a down-regulated miRNA in mouse steatohepatitis model, led to suppressed activation and proliferation of HSCs with concomitant decrease in expression of Wnt1, Wnt5a, α-SMA, and Col-1 [39]. miR-101 promotes transformation of activated HSCs back into their quiescent forms and suppressed expression of TGF-β1 [76]. Furthermore, ectopic expression of miR-101, which is down-regulated in activated HSCs, greatly reduced CCL_4_-induced hepatic fibrosis and miR-101 inhibitor showed an opposite effect with increased fibrogenesis [76]. miR-200a is down-regulated in activated HSCs and its over-expression *in vitro* significantly inhibited α-SMA activity and further affected the TGF-β1-dependent activation of HSC [88]. This group identified β-catenin and TGF-β2 as the two functional downstream targets of miR-200a [88].

The role of miR-454 has been studied in LX-2 cells treated with TGF-β1 and in liver infected with Schistosoma japonicum in mice [92]. miR-454 was found to be down-regulated in TGF-β1 treated LX-2 cells and in mice infected with Schistosoma japonicum [92]. Overexpression of miR-454 inhibited HSC activation by directly targeting Smad4 but did not have any effect on cell cycle or proliferation [92].

### 5.2. microRNAs Regulate HSC Apoptosis and Proliferation

miR-29, an anti-fibrogenic miRNA, is down-regulated in fibrogenesis and enhancing its expression led to decreased HSC proliferation and increased HSC apoptosis in LX-2 HSC, LX-1, and HSC-T6 cell lines [67,94]. miRNA-146a is down-regulated in HSCs in response to TGF-β1 stimulation and restoration of its expression suppressed TGF-β1-induced HSC proliferation and increased HSC apoptosis [82]. miR-16 is down-regulated in activated HSCs and enhancing its expression demonstrated reduced expression of cyclin D1 (CD1), which in turn promoted cell-cycle arrest and normal apoptosis in HSCs and diminished their proliferation [65]. miR-126* is also down-regulated in activated HSCs and restoring their expression led to decreased proliferation with down-regulation of vascular endothelial factor (VEGF) [79]. Dai *et al.*, demonstrated down-regulation of miR-155 in liver and sera in patients with cirrhosis and by enhancing its expression they were able to inhibit EMT and ERK 1 pathways and induced HSC apoptosis [85]. miR-370 is down-regulated in fibrotic livers in rats and in TGF-β1 stimulated HSCs and its enhancement suppressed HSC activation and proliferation by inducing cell apoptosis through direct binding of miR-370 to the 3′ UTR of the Smoothened (SMO) gene [91].

miR-150 and -194 are down-regulated in HSCs isolated from rats with liver fibrosis [83]. Over expression of these miRNAs significantly reduced HSC proliferation and reduced α-SMA and collagen I levels compared to control [83]. Using bioinformatics analysis, C-myb and rac 1 were found to be the targets of miR-150 and -194, respectively. The authors concluded that these miRNAs inhibited HSC activation and ECM production via in part by inhibiting the expression of these targets [83]. Moreover, over-expression of miR-150 *in vitro* resulted in inhibition of cell proliferation, reduction of ECM proteins, and α-SMA, and this was regulated via targeting expressions of SP1, a mediator of α-1 collagen (Col1A1) expression and Col4A4 [84].

In addition to their antiviral effect, interferons (IFNs) are shown to have anti-fibrotic effects by diminishing HSC activation through reduction of TGF-β1 expression [98,99,100]. Sekiya *et al.*, explored the pathophysiology of IFN activity on HSCs in relation to miRNAs and discovered IFN-β induced miR-195 expression, which in turn inhibited cell proliferation by delaying their G1 to S phase cell cycle progression via down-regulation of cyclin E1 and up-regulation of p21 [86].

Guo *et al.*, validated expression of miR-16, -15b, -122, -138, -143, and -140 and by using a gene ontology bioinformatics they identified anti-apoptotic gene Bcl-2 as the target of miR-16 and -15b. By restoring the expression of these two miRNAs they reduced Bcl-2 expression with subsequent increased expression of apoptotic proteins caspase 3, 8, and 9 thus enhancing apoptosis of activated HSCs [42]. Hepatic fibrosis, which was once thought to be an irreversible process, is now shown to be reversible through HSC apoptosis [28]. It is therefore critical to understand the mechanism of action in which miRNAs drive HSC apoptosis as they may be used as potential therapeutic intervention.

### 5.3. microRNAs Regulate HSC Migration

Quiescent HSCs are normally contained within the space of Disse by basement membrane-like matrix but during fibrogenesis, this membrane is lost and is replaced by fibril-forming matrix allowing HSCs to migrate and deposit ECM [101]. miR-31, an up-regulated miRNA during fibrogenesis, play a role in HSC activation and its inhibition diminished HSC activation and migration [54]. miR-335 expression is significantly reduced during HSC activation and by restoring its expression there was decreased HSC migration [90]. Restoration of miR-335 expression inhibits HSC migration via down-regulation of tenascin-C (TNC), an extracellular matrix glycoprotein involved in cell migration [90].

### 5.4. microRNAs Regulate ECM Deposition and Maturation

Over-expression of miR-29 leads to decreased collagen deposition *in vitro* via suppression of alpha-1 type I collagen (Col1A1) gene expression HSC cell lines [67,70]. miR-29 also plays a role in post-translational processing of ECM and fibril formation. Zhang *et al.* showed an inverse correlation between miR-29b expression and heat shock protein 47 (HSP47) and lysyl oxidase, which are essential regulators of ECM maturation and demonstrated overexpression of miR-29b led to abnormal collagen formation [73]. miR-335 expression is significantly reduced during HSC activation and by restoring its expression there was reduced production of α-SMA and collagen type I [90]. Chen L *et al.*, revealed connective tissue growth factor (CCN2), involved in fibrogenesis, is inversely regulated by miR-214 at an epigenetic level [89]. They demonstrated CCN2 expression increased in HSC by fibrosis-inducing stimuli through binding of miR-214 on the CCN2 3′-UTR in an exosome-dependent regulation [89]. They later demonstrated that E-box in the miR-214 promoter binds to basic helix-loop-helix transcription factor, Twist1, which in turn suppressed CCN2 expression. Furthermore, they showed high levels of Twist1 in nanovesicular exosomes secreted by quiescent HSCs and not by activated HSCs, and determined exosomal Twist1 was transported between HSCs and stimulated expression of miR-214 [62]. miR-126* is also down-regulated in activated HSCs and restoring their expression led to decreased ECM production and cell contraction while negatively regulating vascular endothelial factor (VEGF) [79]. miR-122 is another miRNA down-regulated in activated HSCs. There was reduced collagen maturation and ECM production when miR-122 was overexpressed via suppression of prolyl 4-hydroxylase subunit alpha-1 (P4HA1) expression [77]. Similarly, miR-133a is down-regulated in activated HSCs and its over-expression in murine HSC led to decreased expression of collagen [80]. miR-19 decreases following hepatic injury and exogenously enhancing its levels were associated with decreased collagen production [43].

### 5.5. miRNAs Involved in Nonalcoholic Steatohepatitis

Non-alcoholic steatohepatitis (NASH) is a type of hepatic inflammatory process caused by hypercholesterolemia and Tomita *et al.*, demonstrated miR-33a, a miRNA involved in cholesterol metabolism, plays a role in HSC activation in NASH mouse model [56]. They showed increased fat intake accelerated hepatic fibrosis in mice via accumulation of free cholesterol (FC) in HSC, which made them sensitized to TGF-β1-induced activation. FC accumulation in HSCs was enhanced via miR-33a signaling through the suppression of peroxisome proliferator-activated receptor γ (PPARγ) signaling along with HSC activation and disruption of SREBP2-mediated cholesterol-feedback system in HSCs [56].

### 5.6. miRNAs Studied in Cholestatic Mouse Model

Cholestatic mouse model is performed by ligating the bile duct of the mice. Tiao *et al.*, studied miR-29 in cholestatic mouse model where they performed bile duct ligation in miR-29a transgenic (miR-29a Tg) and wild-type (WT) mice to elucidate the role of miR-29 in cholestatic liver injury [72]. They demonstrated improved liver function in miR-29a Tg mice compared to WT following bile duct ligation. They showed decreased expression of collagen-1α1, collagen-4α1, phosphor-FADD, cleaved caspase-8, cleaved caspase-3, Bax, Bcl-2, poly ADP ribose polymerase (PARP), and nuclear factor-κB (NF-κB) and up-regulation of phospho-AKT expression following overexpression of miR-29a.

Lu *et al.*, studied rat liver fibrosis model using CCL_4_ and common bile duct ligation and demonstrated overexpression of miR-130a and -130b. They further demonstrated overexpression of miR-130a and -130b decreased PPARγ expression by targeting the 3′-UTR of PPARγ mRNA in rat HSC-T6 cell lines [60].

### 5.7. miRNAs in in vivo Portal Hypertension Model

Qi *et al.*, tried recapitulating the effect of portal hypertension on hepatic stellate cells by exposing primary rat HSCs to static water pressure of 10 mmHg for 1 h and demonstrated up-regulation of miR-9a-5p [49]. Furthermore, they showed miR-9a-5p suppressed proliferation, migration, and activation of HSCs via down-regulation of Sirt1 [49].

### 5.8. Other Regulatory Mechanisms

miR-19b is found to be down-regulated in fibrotic liver in rats and humans and there was diminished expression of TGF-β1 and subsequent abrogation of fibrogenesis in HSC transfected with miR-19b mimic [43]. Ge *et al.*, studied the potential protective effect of estradiol on fibrogenesis in relation to miRNAs and found miR-19b reduced HSC proliferation by targeting growth factor receptor-bound 2 (GRB2) in hepatic fibrosis models *in vivo* and *in vitro* as part of an inhibitory effect of estradiol [66]. They demonstrated inverse relationship between miR-19b and GRB2 expression where miR-19b was down-regulated and GRB2 mRNA was up-regulated in rat fibrosis model with reversal of these effects in estradiol treated rats [66].

RNase III (Dicer), a key enzyme in miRNA processing, appears to play a role in HSC activity [102]. Yu *et al.*, demonstrated by inhibiting the expression of Dicer, they were able to reduce HSC proliferation and decreased expression of fibrosis-related genes (e.g., type I collagen, α-SMA, and tissue inhibitor metalloproteinases) [102]. During Dicer inhibition, there was significantly decreased expression of miR-138, -143, -140, and -122 and they further elucidated their targets as PTEN, Ras GTPase activating-like protein 1 (RASAL 1), acyl-Coa synthetase long-chain family member 1 (ACSL 1), and p27, respectively [102]. This study emphasized the importance of Dicer-mediated miRNA regulation and its effect on fibrogenesis.

EMT is involved in the activation of HSCs and Yu *et al.*, studied miRNA-mediated epigenetic regulation of EMT [103]. They studied Salvianolic acid B (Sal B), a compound reported to ameliorate oxidative damage by reducing accumulation of reactive oxygen species in hepatocytes, and they demonstrated its anti-fibrogenic effects were in part caused via inhibition of EMT and the Hedgehog (Hh) pathway. They demonstrated Sal B decreased DNA methylation via up-regulation of miR-152, which directly targeted DNA methyltransferase 1 (DNMT1) [103].

## 6. microRNA as a Potential Therapeutic Target in Liver Fibrosis

miR-29 is the most extensively studied miRNA in the pathophysiology of liver fibrosis [69,73]. Knabel *et al.*, studied systemic injection of miR-29a expressing adeno-associated virus (AAV) in mice and demonstrated reversal of histologic and biochemical evidence of hepatic fibrosis despite continued exposure to CCL_4_ and they concluded that AAV-mediated restoration of miR-29a could prevent development of liver fibrosis [104]. Furthermore, nuclear receptor farnesoid I receptor (FXR), a receptor expressed in HSCs and a negative regulator of HSC activation with potent anti-fibrotic activity, is shown to target miR-29a. Li *et al.*, demonstrated, by using a synthetic FXR ligand, there was up-regulation of miR-29a and subsequent decrease in mRNA expression of genes responsible for ECM production [105].

By establishing an artificial intronic miRNA expression system, Chang *et al.*, were able to successfully produce mature anti-TGF-β1 miRNAs, which in turn inhibited expression of TGF-β1 and decreased the expression of their downstream targets such as collagen I, MMP2, TIPM-1, *etc.*, which abrogated development of fibrosis [106].

miR-21 is up-regulated during fibrogenesis. 3,3′-Diindolylmethane (DIM), a natural autolytic product in plants that down-regulates miR-21, decreased HSC activation and attenuated liver fibrosis induced by thioacetamide [107]. Curcumin (*i.e.*, diferuloylmethane, C21H20O6) a polyphenol isolated from yellow curry pigment of turmeric has previously shown to inhibit HSC activation [108]. Zheng *et al.*, studied the mechanism in which curcumin acted on HSCs and discovered it regulated miRNA-mediated control of DNA methylation and controlled fibrogenesis at an epigenetic level via up-regulation of PTEN [74]. This study demonstrated significant up-regulation of miR-29b with curcumin treatment with reduced DNA methyltransferase 3b (DNMT3b) and hypomethylation of PTEN [74].

miR-122 is another miRNA that has been studied as potential systemic therapy for treatment of hepatic fibrosis [78]. Its levels are down-regulated in CCl_4_-induced liver fibrosis models in mice and in HSCs treated with TGF-β1. Restoration of miR-122 was associated with decreased expression of α-SMA, fibronectin 1 (FN1), and COL1A1 by inhibiting the expression of serum response factor (SRF), a key transcription factor that mediates activation of fibrogenic cells [78]. Systemic injection of miR-122-expressing lentivirus increased miR-122 level and reduced the amount of collagen fibrils, FN1, and SRF in the livers of CCl_4_-treated mice [78].

Although not specific to hepatic stellate cells and fibrosis, a great example of use of miRNA that has been applied in clinic as potential therapeutic target is miR-122. There has been a Phase 2a study utilizing Miravirsen, a locked nucleic acid–modified DNA phosphorothioate antisense oligonucleotide that sequesters mature miR-122 [109]. HCV virus relies on miR-122 for their replication and by injecting Miravirsen, there was prolonged dose-dependent reduction in HCV RNA levels without any long-term safety issues [109,110]. miRNAs responsible for fibrogenesis may be targeted in a similar manner to ameliorate and reverse the effect of hepatic injury and fibrosis.

## 7. microRNA as a Potential Biomarker of Liver Fibrosis

The levels of miRNA in serum are stable and reproducible and can be reliably detected and quantified and they have been the subject of much research as potential biomarker for various disease processes including cardiovascular disease and cancers [111,112,113]. It has been shown that circulating miRNAs in plasma come in 2 main forms: bound to RNA-binding protein and lipoprotein complexes or packaged into microparticles (e.g., exosomes, microvesicles) [114].

miR-122 is the most abundant miRNA in hepatocytes and it plays a role in various functions in the liver, including lipid metabolism and hepatic development, and they also have functions in response to hepatic injury [115]. Their use as potential biomarkers is the most studied and published. Most studies are done in the context of viral hepatitis, NASH/NAFLD, and HCC and not specific to fibrogenesis [116,117,118,119,120]. Cermelli and colleagues demonstrated elevated serum levels of miR-122 and -34a correlated with disease severity and fibrosis stage [117]. They concluded that serum miR-122 and -34a may represent novel biomarker to test for disease severity in patients with HCV and NAFLD. However, Bihrer *et al.*, demonstrated serum miR-122 correlated with hepatic transaminase levels but did not show correlation with degree of fibrosis or with synthetic functions of the liver, such as international normalized ratio (INR) and serum albumin levels [116]. Furthermore, some data suggest inverse correlation between circulating and hepatic miR-122 levels with disease severity [61,120]. For example, Trebicka and colleagues demonstrated lower levels of miR-122 in both liver and serum in patients with HCV hepatitis with inverse correlation with disease severity [120]. Morita *et al.*, also showed inverse correlation of hepatic miR-122 levels with severity of liver disease [61]. Given these discordant findings, the utility of miR-122 as a biomarker needs to be further elucidated on larger sample size and on samples from different etiologies of hepatic fibrosis before it can be applied in clinical setting.

miR-181b is elevated in serum of patients with liver cirrhosis. Wang and colleagues showed TGF-β1 induced miR-181b expression and this in turn increased HSC proliferation via regulation of cell cycle by targeting p27 gene [121]. miR-133a levels are also increased in serum of patients with chronic liver disease and their levels correlated with extent of hepatic disease and progression [122]. Similarly, miR-17-5p, a regulator of TGF-β/Smad pathway, was up-regulated in CCL_4_-induced hepatic fibrosis models in mice and was also elevated in serum of patients with cirrhosis compared to healthy controls [50]. miR-33a levels were studied in hepatic tissue and in serum of patients with chronic hepatitis B (HBV) infection [123]. It was elevated in serum of patients with HBV and its levels correlated with degree of fibrosis [123]. miR-122, -33a, -181b, miR-133a, and miR-17-5p, therefore, may potentially serve as a biomarker for diagnosis of liver fibrosis and markers for disease progression [80,117,121,122].

Chen *et al.*, demonstrated miR-214 in circulating exosomes in mice with liver fibrosis, which reflected fibrosis-induced changes in the liver thus highlighting the use circulating exosomes as potential novel biomarker for liver fibrosis [62]. Balas *et al*. further studied circulating miRNAs in association with exosome-rich and protein-rich compartments and their levels varied depending on the etiology of hepatic injury [124]. For example, in liver injury caused by inflammation and alcohol, it was associated with elevated miR-122 and miR-155 predominantly associated with exosome-rich fraction, while in toxin-induced liver injury these miRNAs were predominantly present in the protein-rich fraction [124]. This may be utilized as biomarkers to differentiate the etiology of injury and this finding further emphasizes the complexity of cellular response to different types of hepatic injury.

Levels of certain circulating miRNAs may be used as prognostic markers in cirrhotic patients with portal hypertension [125,126]. Jansen *et al.*, sampled blood from portal and hepatic veins in cirrhotic patients undergoing transjugular intrahepatic portosystemic shunt (TIPS) procedure and showed higher levels of miR-34a in hepatic veins. Although its levels did not correlate with portal pressure, it correlated with the hepatic congestion index. Furthermore, miR-34a levels correlated with other indices of hepatic function, indicating its potential use as a prognostic biomarker in patients undergoing TIPS [125]. A study by the same group also demonstrated miR-122 is elevated in patients with portal hypertension co-infected with HCV and HIV. Its levels correlated with other measures of hepatic dysfunction (e.g., elevated hepatic transaminases) and they demonstrated inverse correlation between miR-122 levels and hepatic venous pressure gradient [126].

Circulating levels of miRNAs have also shown to correlate with level of hepatic injury in patients with HIV infection [126,127]. For example, Anadol *et al.*, tested serum miRNA levels in patients with HIV, HCV, and HBV infection and demonstrated high levels of miR-122 and -34a correlated with HIV/HCV co-infection, whereas highest miR-22 levels were seen in patients with HIV/HBV co-infection [127]. Circulating levels of miR-122 correlated with the degree of hepatic fibrosis and circulating levels of miR-34a correlated with patients’ history of illicit drug and alcohol use [126,127].

## 8. Conclusions

According to the Centers for Disease Control and Prevention (CDC) there were approximately 101,000 patients who were discharged from the hospital with primary diagnosis of cirrhosis or liver disease in 2010 and there were approximately 36,000 death related to their complications in 2013 in the United States [128]. Development of cirrhosis can be prevented by cessation and/or treatment of the inciting liver-injuring event, however once cirrhosis ensues there is currently no effective treatment to reverse the process of hepatic fibrosis.

Hepatic stellate cells are increasingly being recognized as potential therapeutic targets for treatment of hepatic fibrosis [2,4,35,37]. Their activation leads to a pro-fibrotic state with release of pro-inflammatory cytokines and deposition of collagen and extracellular matrix. Moreover, their apoptosis is related to reversal of fibrosis. There is increasing evidence that miRNAs play essential regulatory effects on HSCs with multiple miRNAs that are dysregulated in activated HSCs (Table 2 and Table 3). They regulate activation of HSCs from their quiescent forms, induce HSC proliferation, and they regulate expression of genes involved in collagen production and deposition of extracellular matrix. Additionally, they regulate cell cycle progression and apoptosis of HSCs. Furthermore, there are miRNAs that control the expression of TGF-β1, a potent pro-fibrotic cytokine. Therefore miRNAs have regulatory effects on fibrogenesis at multiple different levels. Altering expression levels of several miRNAs have shown to alter fibrogenesis in both *in vitro* and *in vivo*. As well, there are very promising studies demonstrating systemic delivery of miRNAs via viral vectors with resultant abrogation of fibrogenesis. In addition to being a novel therapeutic target, some miRNAs can potentially be used as biomarkers as some are elevated in serum in patients with liver disease. Furthermore, their levels correlated with degree of liver disease, therefore, they may serve as potential prognostic markers and/or as surveillance markers of disease progression.

Currently, there are 265 clinical trials involving miRNAs but majority of them are observational studies looking at their levels in response to therapy or studying them as biomarkers [129]. One great example of miRNA being uses as a potential therapeutic agent is clinical trial on anti-miR122 (Miravirsen) in treatment of HCV genotype 1 which relies on miR-122 for replication [109]. The use of Miravirsen has shown prolonged reduction of HCV RNA in a dose-dependent fashion [109]. Although there are currently no human clinical trials involving miRNA and HSCs, there is promising data both *in vitro* and *in vivo*.

There are currently 127 studies published on PubMed under the search hepatic stellate cells and microRNA. This is an area of much research with tremendous amount of new information being published on a monthly basis and there are numerous miRNAs in activated HSCs that play a role in fibrogenesis. However, as noted in this review, there is an enormous heterogeneity and variability in the miRNA profile depending on the studies. This heterogeneity could be explained by the use of different species in their study (rodents *versus* humans), *in vitro versus in vivo* activation of HSC, and different etiologies of HSC activation (CCl_4_, bile duct ligation, *etc.*). Animal models provide valuable information and enable us to test *in vivo* hepatic injury model, however, their response to hepatic injury and their miRNA profiles may not necessarily mirror that of humans. Furthermore, we need to note the pitfalls in using miRNAs as potential biomarkers, as there is currently no standardized protocol for sample normalization and sample collection/processing [122]

.miRNAs have shown to play a pivotal role in hepatic fibrogenesis and their potential use as therapy and biomarkers are very promising. However, there needs to be standardization in research protocol and, additionally, their function and mechanism of action need to be further studied in order to translate them into clinical settings.

## Figures and Tables

**Table 1:** Expression Profiling of Differentially Exressed microRNAs in Activated Stellate Cells.

Ref.	miRNA Detection Method	Species	Experimental Model	Number of Significantly Dysregulated miRNAs	Upregulated miRNAs	Downregulated miRNA	Examples
[47]	Microarray	Human	Biopsy samples from individuals with NASH	46	23	23	miR-34a, -122, -146b, -451
[39]	Microarray	Mouse	NASH model using methionine-choline deficient diet	37	19	18	miR-146a-5p
[41,42]	Microarray	Rat	Culture activated	21	12	9	miR-15b, -16
[43]	Microarray	Rat	Culture activated	55	NR	NR	miR-19a, -19b, -92a
[40]	HT sequencing	Human	Biopsy samples from individuals with NAFLD	75	30	45	miR-182
[44]	Microarray	Rat	Culture activated	42	16	26	miR-26a, -29a, -214
[45]	Microarray	Mouse	CCL_4_-induced fibrosis	NR	11	NR	miR-200a, -200b
[46]	Microarray	Mouse	CCL_4_-induced fibrosis	31	10	21	miR-29

NR = not reported, HS = high throughput, NAFLD = nonalcoholic fatty liver disease, NASH = nonalcoholic steatohepatitis.

**Table 2:** Up-regulated miRNAs in activated hepatic stellate cells.

miRNA	Target Genes	Potential Roles	References
miR-9a-5p	Sirt1	Proliferation and migration	[49]
miR-17-5p	Smad7	HSC activation and proliferation	[50]
miR-21	PTEN, AP-1, SPRY2, HNF4α, PDCD4	Collagen synthesis and HSC activation	[51,52,53]
miR-31	FIH1	Activation, proliferation, and migration	[54]
miR-33a	PPARΥ	HSC activation	[55,56]
miR-34a and -34c	PPARΥ, ACSL1	HSC activation	[57,58]
miR-126	IκBα	HSC activation and TGF-β expression	[59]
miR-130a and -130b	PPARΥ	HSC activation and proliferation	[60]
miR-181b	p27, PTEN	Cell proliferation	[61]
miR-214	CCN2	HSC activation	[62]
miR-214-5p	MMP-2, MMP-9, α-SMA, TGF-β1	HSC ctivation	[63]
miR-221 and -222	CDKN1B	Collagen deposition	[64]

**Table 3:** Down-regulated miRNAs in activated hepatic stellate cells.

miRNA	Target Genes	Potential Roles	References
miR-16	CD1	Apoptosis	[65]
miR-19b	GRB2, TGF-β1	HSC proliferation	[43,66]
miR-29	IGF-I, PDGF-C, HSP47, Col1A1	Collagen expression and cell proliferation	[67,68,69,70]
miR-29a	HDAC4, Various ECM producing genes	HSC activation and ECM production	[71,72]
miR-29b	HSP47, Col1A1, SP1, PTEN	Collagen production and maturation	[73,74]
miR-30	KLF11	TGF-β1 expression	[75]
miR-101	TβRI, KLF6	Fibrinogenesis, proliferation, and migration	[76]
miR-122	P4HA1, FN1, SRF,	Collagen production, maturation and ECM production	[77,78]
miR-126*	VEGF	Proliferation and cell contraction	[79]
miR-133a	ECM	Collagen production	[80]
miR-144	TGF-β1	HSC activation	[81]
miR-146a	SMAD4, Wnt1, Wnt5a	Proliferation and proliferation	[39,82]
miR-150	c-myb, SP1, Col4A4	HSC activation and collagen production	[83,84]
miR-155	TCF4 and AGTR1	HSC activation	[85]
miR-195	cyclin E1, p21	Cell proliferation	[86]
miR-200a	α-SMA, β-catenin, TGF-β2, Keap1	HSC activation and proliferation	[87,88]
miR-214	CCN2	Hepatic fibrosis through α-SMA	[89]
miR-335	TNC	HSC migration, production of α-SMA, and collagen type I	[90]
miR-370	SMO	Proliferation	[91]
miR-454	Smad4	HSC activation	[92]
miR-483	PDGF-β, TIM2	HSC activation	[93]
